# Integration of health services, access and utilization by refugees and host populations in West Nile districts, Uganda

**DOI:** 10.1186/s13031-018-0184-7

**Published:** 2019-01-07

**Authors:** Henry Komakech, Lynn Atuyambe, Christopher Garimoi Orach

**Affiliations:** Department of Community Health and Behavioural Sciences, Makerere University School of Public Health, P. O. Box 7072, Kampala, Uganda

**Keywords:** Uganda, Refugees, Access, Utilization, Healthcare, Integration

## Abstract

Prolonged civil war and unrest has dominated the short history of South Sudan resulting in the long-term displacement of millions of people since the 1990s. Since December 2013, over one million South Sudanese refugees have fled into Uganda. International and national responses to the refugee influx in the country has been managed through parallel and integrated care and assistance mechanisms. In order to enhance effectiveness and responsiveness of assistance programmes to refugees and host communities in the country, governments and aid agencies should promote the integration of services.

## Background

Since 15th December 2013, South Sudan has been going through a civil war. The conflict has resulted to the displacement of over 3.1 million inhabitants. About 2.4 million refugees live in neighbouring countries including; 785,104 in Uganda, 768,125 in Kenya, and 445,441 in Ethiopia [1]. In Uganda, the majority, 71% of refugees originate from South Sudan. The refugees live in 23 settlements across five districts including; 271,655 in Arua, 257,104 in Adjumani, 151,304 in Moyo, 286,895 in Yumbe and 4623 in Koboko district. About 33% of the population in these districts are refugees [2]. Globally, this is one of the highest ratio of refugees to host population.

Health services for refugees and host populations are provided based on an integrated approach. The integration process is guided by the Uganda National Integrated Response Plan for Refugees and Host Communities and the Global Strategy for Public Health 2014–2018 of the United Nations High Commission for Refugees (UNHCR). These plans operationalize integration by linking humanitarian and development programming and interventions. Service delivery is aligned with the National Health Policy and Health Sector Development Plan 2015/6–2019/20 [3]. Health services integration is promoted and implemented by the Ministry of Health (MoH) through the district health system. Aid agencies support the district health services to enable refugee’s access healthcare. Implementation follows a four pronged process including; accreditation and alignment of health facilities and workers by MoH standards, capacity strengthening, supporting coordination and leadership within MoH and districts, and realigning aid agencies to support health service delivery [4].

Health services in the west Nile districts are provided by public and private health facilities [4]. In Arua and Adjumani districts, health services are provided through public facilities with support from the UNHCR. While in Moyo, Koboko and Yumbe districts, health service are provided by the UNHCR through aid agencies [5]. Service delivery has expanded through establishment of health facilities by the UNHCR [6]. Access to health facilities (within 5 km) varies between 16 and 36% in Adjumani, 24–68% in Arua, 16% in Koboko, 19% in Moyo and 35–65% in Yumbe district.

**Fig. 1:**
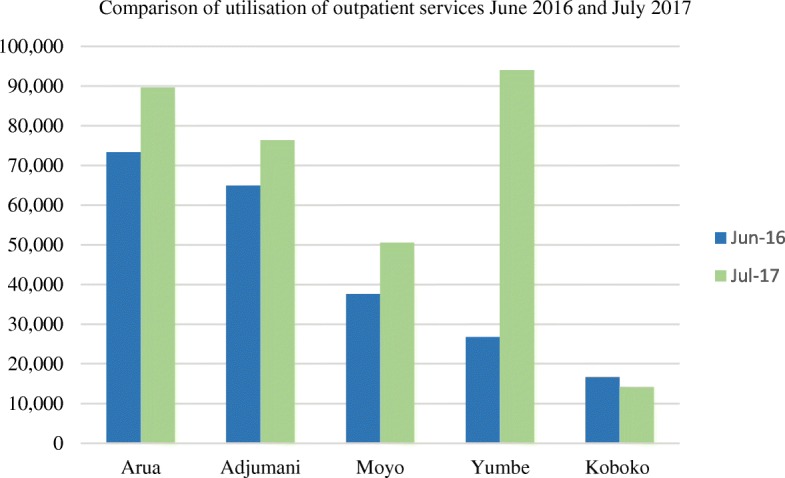
Utilization of outpatient services by refugee and host populations during June 2016 and July 2017

Between January 2014 and June 2017, populations served by health facilities increased from 21 to 36% in Adjumani, 24–68% in Arua and 35–65% in Yumbe districts. Institutional deliveries increased from 63 to 90% in Adjumani, 40–96% in Arua and 68–100% in Yumbe districts between June 2016–July 2017[7]. Outpatient consultations increased from 20 to 70% in Adjumani, 12–77% in Arua and 50–65% in Yumbe districts during June 2016–July 2017 [7]. About 22% (1,557,987) outpatient consultations were made by host communities. [8] (Fig. 1).

## Conclusion

Public health response to population displacements in developing countries remains a challenge. Health service delivery is affected by weak health systems due to inadequate funding, low human resource capacity and a high disease burden. Humanitarian assistance is critical towards improving the wellbeing of the displaced and host populations. It is therefore essential to promote the integration of health services in order to improve equity, effectiveness and efficiency of health services delivery.

## Abbreviations

CSBAG: Civil Society Budget Advocacy Group
JICA: Japan International Cooperation Agency
MoH: Ministry of Health
RMF: Real Medical Foundation
UNHCR: United Nations High Commission for Refugees

## References

[1] UNHCR (2018). South Sudan situation, United Nations high Commision for refugees.

[2] UNHCR (2018). Uganda Comprehensive Refugee Response Portal.

[3] MoH (2015). Health Sector Development Plan 2015/6–2019/20. M. o. Health.

[4] UNHCR (2018). Health and nutrition, Uganda.

[5] RMF (2018). Wdespread medical needs addressed in Bidibidi refugee settlement, Ream Medical Foundation.

[6] CSBAG (2018). Public financing for the refugee crisis in Uganda.

[7] JICA (2018). Situation Assessment and Needs Identification presentation for Health sector - West Nile Sub-region - 27 Feb 2018.

[8] UNHCR (2017). Public health annual report 2017.

